# Association between Undernutrition at Admission and Improvement in Balance Function Post-stroke

**DOI:** 10.31662/jmaj.2024-0228

**Published:** 2024-11-01

**Authors:** Keisuke Sato, Seiji Tanaka, Masaki Koike, Takahiro Ogawa

**Affiliations:** 1 LIM PROJECTS Inc., Nakagami, Japan; 2 Chuzan Hospital Clinical Education and Research Center, Okinawa, Japan; 3 Department of Rehabilitation Medicine, Aichi Medical University Graduate School of Medicine, Nagakute, Japan; 4 Department of Rehabilitation Medicine, Aichi Medical University, Nagakute, Japan; 5 Kobe College of Medical Welfare, Mita, Japan

**Keywords:** stroke, balance function, rehabilitation, undernutrition

## Abstract

**Introduction:**

The prognosis for activities of daily living (ADL) ability after stroke is negatively influenced by undernutrition and impaired balance. However, the association between undernutrition and balance improvement has not yet been elucidated. This study aimed to investigate the influence of undernutrition on balance function improvement in patients with stroke.

**Methods:**

This retrospective observational study included patients with cerebral infarction aged ≥65 years. The study period was from May 2018 to May 2022. The patients were divided into undernutrition and intact nutrition groups according to the Global Leadership Initiative on Malnutrition criteria. The primary outcome was the change in the Berg Balance Scale (BBS) score (BBS score at discharge − BBS score at admission).

**Results:**

This study included 304 patients (mean age, 79.2 ± 8.1 years; 173 men and 131 women). These patients were divided into the undernutrition (N = 114) and intact nutrition (N = 190) groups. The undernutrition group demonstrated lower BBS scores at admission (16.0 ± 17.1 vs. 28.3 ± 18.4, *p* < 0.001) and at discharge (24.2 ± 19.6 vs. 40.0 ± 16.9, *p* < 0.001) than the intact nutrition group. After adjusting for confounding factors, undernutrition was associated with a smaller change in the BBS score (coefficient = −2.988, 95% confidence interval = −5.481 to −0.495, *p* = 0.019).

**Conclusions:**

Undernutrition negatively influences balance function recovery in post-stroke patients. A strategy aimed at improving nutritional status could have beneficial effects on patients’ balance function.

## Introduction

Recovery of mobility is one of the goals of rehabilitation in patients with stroke. Studies have reported that 15%-30% of patients with stroke have permanent disabilities ^(1)^ and require assistance with activities of daily living (ADL). Physical activity and motor function impairment following stroke may lead to reduced mobility and ADL ability ^(2), (3)^. It is also one of the indicators that determine the outcome ^(4), (5)^. Therefore, restoring mobility and ADL ability is a major challenge for patients with stroke as well as their families and healthcare providers.

Various factors have been reported as predictors of mobility recovery ^(6), (7)^, particularly balance function, which is the most common indicator of mobility in patients with stroke ^(8), (9), (10), (11), (12)^. Balance function is the ability to maintain the body’s center of gravity in and out of the basal plane of support and equilibrium. It is necessary for stable sitting and standing positions. Impaired balance leads to reduced mobility in patients with stroke ^(8), (9), (10), (11), (12)^ and is associated with an increase in falls ^(13), (14), (15)^. Thus, balance is considered to be a necessary function for mobility and ADL. Moreover, balance function has been reported to be a predictor of the length of hospital stay and discharge destination ^(16)^. Therefore, balance improvement is important for restoring mobility and ADL ability in patients with stroke.

Undernutrition is prevalent among patients with stroke undergoing rehabilitation ^(17), (18)^, which negatively affects their ADL ability ^(19), (20)^. Several studies have reported that undernutrition in such patients is negatively associated with longer hospital stay and ADL ability ^(21), (22)^. It has also been reported that undernutrition during admission to an acute or convalescent rehabilitation ward suppresses the recovery of trunk function ^(23), (24)^. Thus, although there have been reports on undernutrition and trunk function, the association between undernutrition and balance function has not yet been elucidated. Meanwhile, sarcopenia is an important factor in the functional recovery of patients with stroke ^(25)^, and its diagnosis requires the measurement of gait speed and grip strength. However, this measurement is difficult in many patients with stroke owing to decreased level of consciousness, cognitive decline, aphasia, and other factors. Therefore, even in patients for whom accurate assessment of sarcopenia is difficult, a more realistic approach is to focus on malnutrition assessment. If undernutrition is associated with fewer changes in balance function, efforts to improve patient nutrition would be promising for improving balance and ADL ability. Therefore, we aimed to investigate the influence of undernutrition on the improvement of balance function in patients with stroke.

## Materials and Methods

### Participants

This retrospective observational study included patients with cerebral infarction aged ≥65 years. The study period was from May 2018 to May 2022. The patients who did not undergo evaluation for cerebral infarction severity, nutritional status, or balance function on admission were excluded.

This study was conducted in compliance with the principles of the Declaration of Helsinki and according to the ethical guidelines of Aichi Medical University Hospital. The study protocol was approved by the Ethical Committee of Aichi Medical University Hospital (approval number: 2023-012). The requirement for informed consent was waived due to the retrospective nature of the study. Therefore, a notice on the website gave participants the option to opt out of the study. Furthermore, they had the option to leave the study at any time.

### Data collection

We extracted data on patient characteristics, including age, sex, type of stroke, history of stroke, history of fractures, body mass index (BMI), Mini-Nutritional Assessment-Short Form (MNA-SF) score ^(26)^, days from stroke onset to rehabilitation hospital, National Institutes of Health Stroke Scale (NIHSS) score (used to assess stroke severity) ^(27)^, Berg Balance Scale (BBS) score (used to assess balance function) ^(28)^, and Functional Independence Measure (FIM) score (used to assess ADL ability), from the patients’ medical records ^(29)^.

The attending physical therapist assessed the severity of stroke using the NIHSS score at admission. A high NIHSS score indicated significant neurological severity, with the total score ranging from 0 to 42. The attending physical therapist and nurse assessed ADL ability using the FIM scores at admission and discharge, which consisted of 13 physical and 5 cognitive items scored on a scale from 1 to 7. The total score ranged from 18 to 126. A low FIM score indicates low independence in ADL. We also determined the total rehabilitation volume (min) from the patients’ medical records and calculated the rehabilitation volume per day (min/day).

### Nutritional assessment

On admission, a nutritionist assessed the patients using the MNA-SF and Global Leadership Initiative on Malnutrition (GLIM) criteria ^(30)^. The application of the GLIM criterion involved a three-step process. Patients were identified in the first stage using a validated screening technique, and phenotypic and etiologic criteria were assessed in the second stage. The final phase involved a severity assessment using phenotypic standards. We used the MNA-SF to screen all patients in this study. The GLIM criteria contain three phenotypic and two etiologic components for diagnosing undernutrition. Phenotypic components included weight loss, low BMI, and reduced muscle mass, whereas etiological components included reduced food intake or assimilation, disease burden, and inflammation. Weight loss was defined as any reduction in body weight of > 5% within the previous 6 months or > 10% beyond that time. A low BMI was defined as a BMI < 20 kg/m^2^ for patients aged < 70 years and < 22 kg/m^2^ for patients aged > 70 years. Reduced muscle mass was defined as a skeletal muscle mass index < 4.75 kg/m^2^ for women and < 6.70 kg/m^2^ for men. The patients were divided into the undernutrition and intact nutrition groups at admission, and patient characteristics were compared between the groups.

### Balance function assessment

The attending physical therapist assessed balance function using the BBS scores at admission and discharge. The BBS is a useful tool for determining balance impairment. The 14 components constituting the BBS score are mainly categorized into the patient’s ability to maintain a static sitting posture (item 3), a static standing position (item 2), and a standing position while moving (items 1 and 4 through 10). A higher BBS score indicates greater balance function. Each of the 14 tasks was graded using a 5-level ordinal scale, with 0 representing “unable to perform or requiring support” and 4 representing “normal performance.” The total score varied from 0 (lowest) to 56 (highest). The primary outcome was the change in the BBS score (BBS score at discharge − BBS score at admission).

### Sample size calculation

Previous studies have demonstrated that the minimal clinically important difference in the BBS was 4 points ^(31)^. Therefore, we calculated the sample size assuming a standard deviation of the population mean of 12.4 points ^(32)^, an alpha error of 0.05, and a power of 0.8 to detect a difference ≥4 points between the groups. Therefore, a sample size of 304 participants was required to reject the null hypothesis.

### Statistical analyses

The median (interquartile range) and mean (standard deviation) were used to define quantitative, including parametric and nonparametric, variables. The Shapiro-Wilk test was employed to determine normality. Categorical variables were expressed as the number of patients (percentage). The chi-squared and Mann-Whitney U tests were employed to evaluate group comparisons for categorical and quantitative data, respectively.

Multiple linear regression analysis was conducted to explore the impact of undernutrition at admission on BBS score changes. We adjusted for age, days from onset to rehabilitation hospital, NIHSS, BBS, and FIM, which have been shown to be associated with balance function in previous studies, as independent variables ^(33), (34)^. The significance level was set at 5%. The R software (version 1.55) was used for all statistical analyses ^(35)^.

## Results

**Figure 1.**
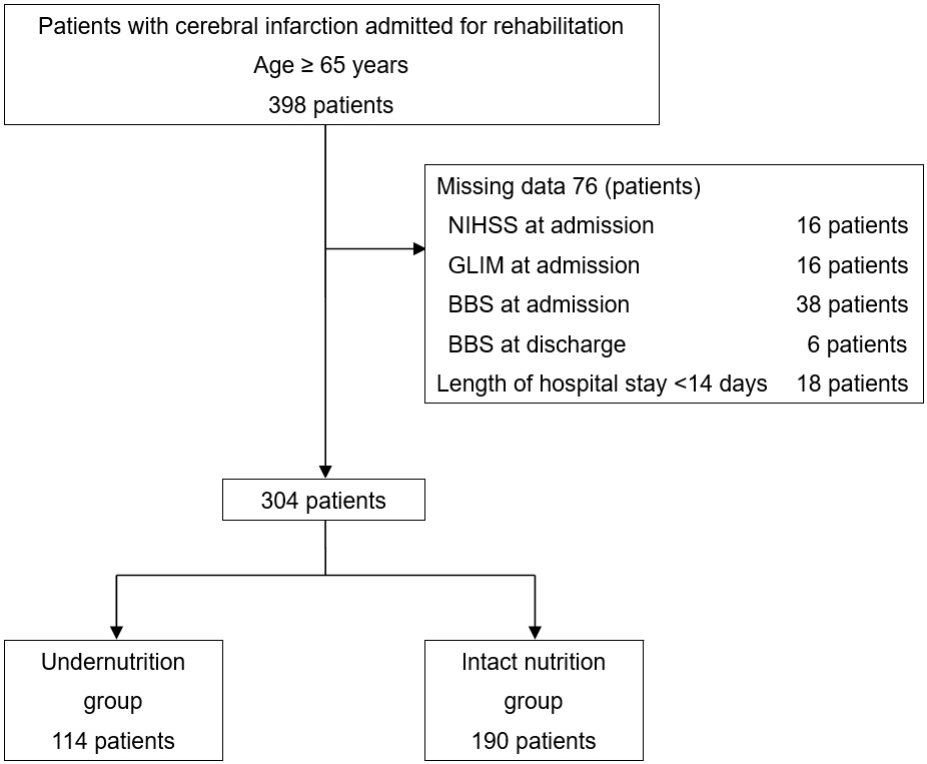
Patient inclusion diagram NIHSS, National Institutes of Health Stroke Scale; GLIM, Global Leadership Initiative on Malnutrition; BBS, Berg Balance Scale.

A total of 398 patients were enrolled in the study, of whom 76 with missing data and 18 with hospital stays of <14 days were excluded. Ultimately, 304 participants were included in the final analysis (Figure 1). The baseline patient characteristics are presented in Table 1. The mean age of the patients was 79.2 ± 8.1 years, and 56.9% were men. The patients were divided into the undernutrition (37.5%) and intact nutrition (62.5 %) groups. The patients in the undernutrition group were older (81.5 ± 8.0 vs. 77.9 ± 7.9, *p* < 0.001) than those in the intact nutrition group. The undernutrition group had higher NIHSS (8 [4-14] vs. 4 [2-8], *p* < 0.001), lower BBS (16.0 ± 17.1 vs. 28.3 ± 18.4, *p* < 0.001), and lower FIM scores on admission (46.0 ± 20.3 vs. 65.6 ± 19.0, *p* < 0.001) than the intact nutrition group. Nutritional status according to the GLIM criteria at admission is presented in Table 2. The most common phenotypic criterion was reduced muscle mass, which was observed in 64.2% and 71.0% of the male and female patients, respectively. In terms of etiological criteria, reduced food intake or assimilation (28.0%) was more common than disease burden/inflammatory conditions (22.4%). The undernutrition group had a significantly higher incidence of low BMI, reduced muscle mass, reduced food intake or assimilation, and disease burden/inflammatory conditions than the intact nutrition group.

**Table 1.** Patient Characteristics at Admission to the Rehabilitation Hospital.

	Overall	Undernutrition group	Intact nutrition group	*p*-value
	(n = 304)	(n = 114)	(n = 190)	
Age, years	79.2 ± 8.1	81.5 ± 8.0	77.9 ± 7.9	<0.001
Sex, n (%)				0.403
Men	173 (56.9)	61 (53.5)	112 (58.9)	
Women	131 (43.1)	53 (46.5)	78 (41.1)
Type of stroke, n (%)				0.001
Lacunar infarction	89 (29.3)	23 (20.3)	66 (34.7)	
Atherothrombotic cerebral infarction	164 (53.9)	61 (53.5)	103 (54.2)	
Cardiogenic cerebral embolism	51 (16.8)	30 (26.3)	21 (11.1)	
History of stroke, n (%)	92 (30.3)	41 (36.0)	51 (26.8)	0.096
History of fracture, n (%)	42 (13.8)	25 (21.9)	17 (8.9)	0.002
BMI, kg/m^2^	23.6 ± 3.8	22.2 ± 3.4	24.5 ± 3.8	<0.001
MNA-SF score, points	8 [6-10]	7 [5-8.8]	9 [7-10]	<0.001
Days from onset to rehabilitation hospital, days	23.0 ± 20.6	27.0 ± 20.2	20.7 ± 20.6	0.010
NIHSS score, points	5 [2-11]	8 [4-14]	4 [2-8]	<0.001
BBS score, points	23.7 ± 18.8	16.0 ± 17.1	28.3 ± 18.4	<0.001
FIM score, points	58.2 ± 21.7	46.0 ± 20.3	65.6 ± 19.0	<0.001

BMI, Body Mass Index; MNA-SF, Mini-Nutritional Assessment-Short Form; NIHSS, National Institutes of Health Stroke Scale; BBS, Berg Balance Scale; FIM, Functional Independence Measure

**Table 2.** Nutritional Assessment of GLIM Criteria Sub-Items at Admission.

	Overall	Undernutrition group	Intact nutrition group	*p*-value
Phenotypic criteria, n (%)				
Weight loss				
>5% within past 6 months	70 (23.0)	48 (42.1)	22 (11.6)	<0.001
>10% beyond 6 months	16 (5.3)	10 (8.8)	6 (3.2)	0.060
Low BMI				
<20 kg/m^2^ if <70 years	5 (10.0)	4 (33.3)	1 (2.6)	0.009
<22 kg/m^2^ if ≥70 years	84 (33.1)	47 (46.1)	37 (24.3)	<0.001
Reduced muscle mass				
Men	111 (64.2)	55 (90.2)	56 (50.0)	<0.001
Women	93 (71.0)	52 (98.1)	41 (52.6)	<0.001
Etiologic criteria, n (%)				
Reduced food intake or assimilation	85 (28.0)	79 (69.3)	6 (3.2)	<0.001
Disease burden/inflammatory condition	68 (22.4)	58 (50.9)	10 (5.3)	<0.001

GLIM, Global Leadership Initiative on Malnutrition; BMI, body mass index

A comparison of the outcomes between the groups is presented in Table 3. The undernutrition group exhibited a lower BBS score at discharge (24.2 ± 19.6 vs. 40.0 ± 16.9) and smaller changes in the BBS score (8.1 ± 8.8 vs. 11.7 ± 11.7) than the intact nutrition group. The rehabilitation volume did not significantly differ between the groups (136.1 ± 21.8 vs. 138.3 ± 20.0). Table 4 presents the changes in the BBS and FIM scores between admission (baseline) and discharge. The BBS score at discharge in all participants was significantly higher than that at baseline (34.1 vs. 23.7, *p* < 0.001), as was the FIM score (86.5 vs. 58.2, *p* < 0.001).

**Table 3.** Outcomes at Hospital Discharge.

	Overall	Undernutrition group	Intact nutrition group	*p*-value
Length of hospital stay, days	82.1 ± 40.6	88.0 ± 41.2	78.5 ± 39.9	0.048
Rehabilitation volume, min/day	137.5 ± 20.7	136.1 ± 21.8	138.3 ± 20.0	0.372
BBS score, points	34.1 ± 19.5	24.2 ± 19.6	40.0 ± 16.9	<0.001
Change in the BBS score, points^*^	10.4 ± 10.8	8.1 ± 8.8	11.7 ± 11.7	0.005
FIM score, points	86.5 ± 31.2	70.3 ± 31.7	96.2 ± 26.6	<0.001
Change in the FIM score, points^†^	28.3 ± 15.9	24.3 ± 16.1	30.7 ± 15.4	0.001

BBS, Berg Balance Scale; FIM, Functional Independence Measure ^*^ The change in the BBS score (BBS score at discharge − BBS score at admission) was calculated. ^†^ The change in the FIM score (FIM score at discharge − FIM score at admission) was calculated.

**Table 4.** Comparison of BBS and FIM between on Admission and at Discharge.

	On admission	At discharge	*p*-value
BBS score, points	23.7 ± 18.8	34.1 ± 19.5	<0.001
Undernutrition group	16.0 ± 17.1	24.2 ± 19.6	<0.001
Intact nutrition group	28.3 ± 18.4	40.0 ± 16.9	<0.001
FIM score, points	58.2 ± 21.7	86.5 ± 31.2	<0.001
Undernutrition group	46.0 ± 20.3	70.3 ± 31.7	<0.001
Intact nutrition group	65.6 ± 19.0	96.2 ± 26.6	<0.001

BBS, Berg Balance Scale; FIM, Functional Independence Measure

**Figure 2.**
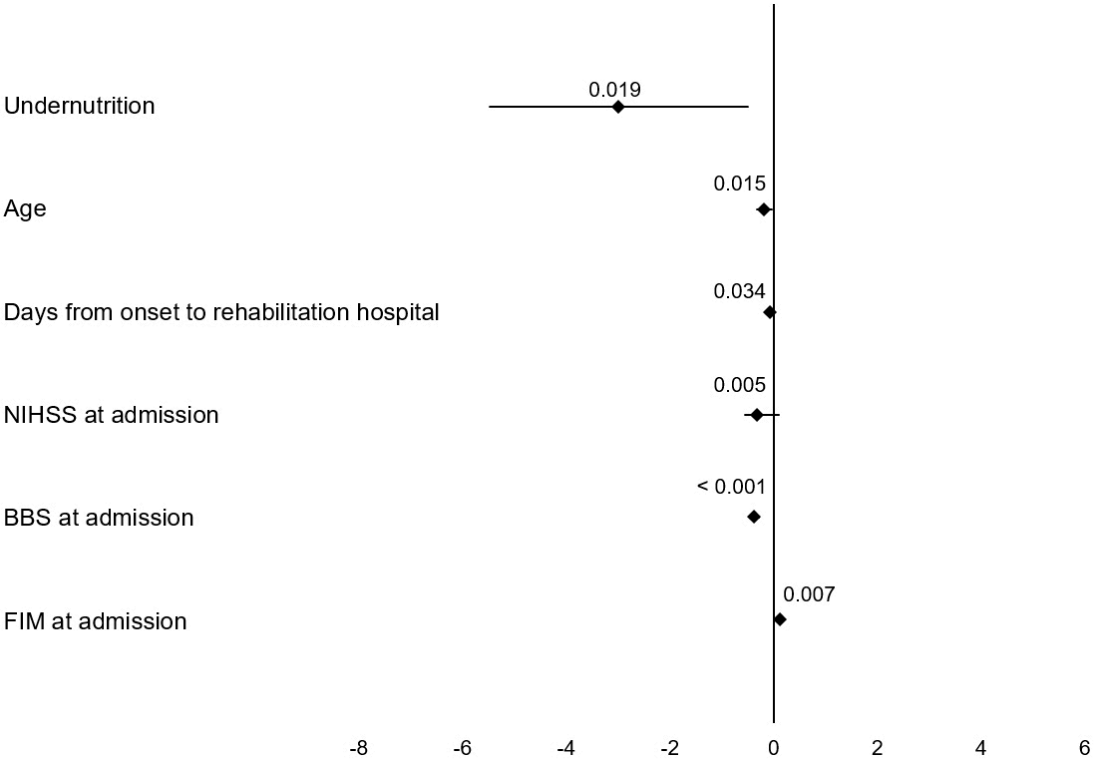
Multiple linear regression analyses for changes in BBS scores Undernutrition was associated with a smaller change in the BBS score (coefficient = −2.988, 95% CI = −5.481 to −0.495, *p* = 0.019). BBS, Berg Balance Scale; NIHSS, National Institutes of Health Stroke Scale; FIM, Functional Independence Measure.

The results of the multiple linear regression analysis of the change in BBS scores are presented in Figure 2. After adjusting for confounding factors, undernutrition was associated with a smaller change in the BBS score (coefficient = −2.988, 95% confidence interval = −5.481 to −0.495, *p* = 0.019).

## Discussion

This study investigated the influence of undernutrition on changes in balance function in patients with stroke and obtained two notable findings. First, undernutrition at admission was associated with reduced improvement in BBS scores. Second, the BBS score at discharge improved compared with baseline in older patients.

Undernutrition at admission was associated with lower BBS score changes in patients with stroke. Several previous studies have examined the association between undernutrition and the risk of falls ^(36), (37), (38)^ and the recovery of trunk function ^(23), (24)^. However, no study has investigated the effect of undernutrition on the recovery of balance function. Several previous studies have reported that the prevalence of undernutrition in patients with stroke is higher in the weeks following stroke onset ^(39), (40), (41)^. It has also been reported that many patients exhibiting undernutrition on admission have reduced skeletal muscle mass and muscle strength ^(42)^. Systematic reviews have indicated that many older adults in hospitals have malnutrition, frailty, and sarcopenia ^(43)^. This suggests that undernutrition limits improvements in skeletal muscle mass and strength, resulting in a smaller recovery of balance function. Nishioka et al. reported that patients with stroke who were underweight showed less improvement in ADL ability than patients with obesity ^(44)^. Furthermore, improved nutrition is associated with the recovery of ADL ability ^(45)^. Therefore, undernutrition on admission to a convalescent rehabilitation ward should be diagnosed at an early stage, and rehabilitation should be performed considering nutritional status. However, a detailed assessment of nutritional intake following hospital admission was not included in this study; thus, it should be considered in the future.

Compared with the baseline, the BBS score improved at discharge. In this study, the patients were aged ≥65 years (mean age: 79.2 ± 8.1 years), and the mean BBS score on admission was 23.7. In previous studies involving post-stroke patients, an increased risk of falls was reported for BBS scores below 42 (mean age: 73 [47-94] years) ^(15)^, 45 (mean age: 69.0 ± 9.5 years) ^(13)^, and 49 (mean age: 68.1 ± 12.8 years) ^(14)^. All participants in this study were older and had lower BBS scores than the threshold for an increased risk of falls in patients with stroke ^(13), (14), (15)^. Previous studies have demonstrated that balance function declines with age ^(33)^. Age-related reduction in skeletal muscle mass and strength has a negative influence on balance ^(46), (47)^. These findings suggest that age-related changes in balance is partly due to a decline in skeletal muscle mass and strength. In addition, several studies have reported that resistance training and nutritional intake in patients with sarcopenia increase muscle mass and strength ^(48), (49)^. Thus, improving skeletal muscle mass and strength while considering nutritional status may help prevent the loss of balance function.

This study has some limitations. First, due to the retrospective nature of the study, we could not fully adjust for the impact of confounding factors on the outcomes. Second, the recovery of balance function in older patients with undernutrition at hospital admission may have been hindered by decreased ADL ability prior to stroke. Third, differences in rehabilitation programs may have affected balance improvement. Moreover, the study did not consider specific details about the types, contents, or intensities of rehabilitation techniques employed, which could be important factors influencing outcomes. Future research should include these variables and use a prospective study design to better understand their effect on rehabilitation effectiveness. Finally, we could not collect data on educational level and socioeconomic status, which could potentially affect nutrition and physical condition. Therefore, we acknowledge that these unaddressed biases may have impacted the study results.

## Article Information

### Conflicts of Interest

None

### Acknowledgement

We would like to acknowledge all the patients who agreed to participate in this study.

### Author Contributions

KS and TO conceived and designed the study. KS performed the data collection and was supported by ST, MK, and TO. KS conducted the data analysis with the support of TO. The initial drafts of the manuscript were written by KS and TO and critically reviewed and edited by the other authors. All authors agreed on the final version of the manuscript and are accountable for all aspects of the work.

### ORCID iD

Takahiro Ogawa: https://orcid.org/0000-0003-4775-5649

### Approval by Institutional Review Board (IRB)

None.
